# Aluminum environmental pollution: the silent killer

**DOI:** 10.1007/s11356-021-14700-0

**Published:** 2021-07-01

**Authors:** Reema H. Alasfar, Rima J. Isaifan

**Affiliations:** Division of Sustainable Development (DSD), College of Science and Engineering (CSE), Hamad Bin Khalifa University (HBKU)/Qatar Foundation (QF), P.O. Box 5825, Doha, Qatar

**Keywords:** Aluminum, Toxicity, Environmental pollution, Alzheimer disease

## Abstract

The concern about aluminum (Al) toxicity has been proven in various cases. Some cases are associated with the fact that Al is a neurotoxic substance that has been found in high levels in the brain tissues of Alzheimer’s disease (AD), epilepsy, and autism patients. Other cases are related to infants, especially premature infants and ones with renal failure, who are at the risk of developing the central nervous system (CNS) and bone toxicity. This risk is a result of infants’ exposure to Al from milk formulas, intravenous-feeding solutions, and possibly from aluminum-containing vaccinations. Furthermore, most antiperspirants contain  aluminum compounds that raise human exposure to toxic Al. This review paper is intended to discuss in detail the above concerns associated with aluminum, and hence urges the need for more studies exploring the effects of overexposure to Al and recommending mitigation actions.

## Introduction

Aluminum, the most abundant metallic element in the earth’s crust, is a light metal with excellent heat and electrical conductivity (Keith et al. 2008). By mass, 8.8% (88 g/kg) of the earth’s crust is aluminum, and it can be found in numerous amounts of rocks (Keith et al. 2008). By the natural weathering of rocks, aluminum is released into the environment (Al-Thani et al. 2018a; Al-Thani et al. 2018b). Air, water, and different kind of foods contain aluminum in their composition (Keith et al. 2008). According to the World Health Organization (WHO), the established tolerable daily intake of aluminum is 1 mg per kg of body weight (Mohammad et al. 2014). However, human bodies are now overexposed to aluminum because of several reasons (Mohammad et al. 2014). Reported studies have shown that ingestion and exposure to high aluminum levels can result in serious health problems (Barabasz et al. 2002). Recently, aluminum is linked to many human diseases, including Alzheimer’s disease (AD) (Mold et al. 2019a; Mold et al. 2019b; Mold et al. 2020). Besides, concerns about the high level of aluminum in milk formulas have questioned the safety of feeding infants with formulas (Chuchu et al. 2013; Igweze et al. 2020; Redgrove et al. 2019). Also, some vaccines contain high concentration of aluminum (Gołoś and Lutynska 2015; Miller 2016a, 2016b). Furthermore, the use of antiperspirants is dermally exposing humans to toxic aluminum (Crisponi et al. 2013).

## Sources of aluminum

**Fig. 1:**
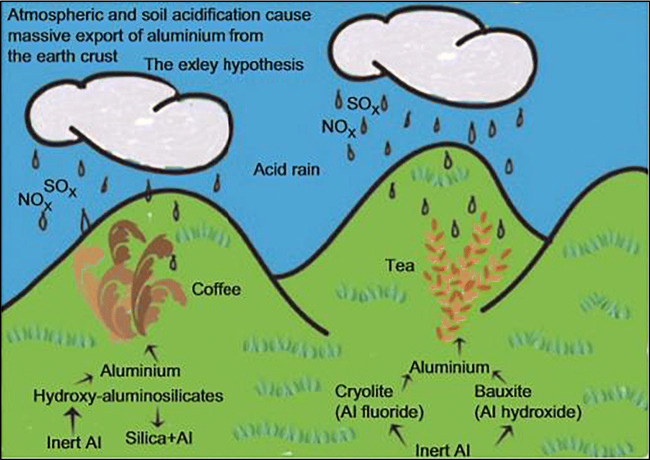
Acid rain releases aluminum into the environment (Crisponi et al. 2013)

The sources of aluminum can be divided into two main parts: natural and anthropogenic sources. Naturally, aluminum exists in the air from the weathering processes as well as from eruptions of volcanoes (Al-Thani et al. 2018b; Mold et al. 2019b). During natural weathering, aluminum is transferred from soil particulates to an aqueous environment (Mohammad et al. 2014). Aluminum’s ability to form organic and mineral complexes with several hydration degrees helps aluminum transfer from solid to liquid phase (soil-water) (Barabasz et al. 2002). Also, since aluminum is highly soluble in an acidic environment, acid rain can cause the amount of dissolved aluminum in the surrounding water to increase (Barabasz et al. 2002; Mold et al. 2019b). In fact, aluminum can exist in several forms in water and is affected by different parameters such as pH which determines the forms of aluminum that are available in an aqueous environment (World Health Organization 2003). Figure 1 shows how acid rain mobilizes aluminum and hence can be a factor for increasing the aluminum level in some plants such as tea leaves and coffee beans (Crisponi et al. 2013). According to Zhou et al. (2020), areas where soils are acid are high in aluminum toxicity and hence constrain plants’ growth.

Some plants have a high concentration of aluminum, such as tea, some kind of herbs and spices, potatoes, and spinach (Barabasz et al. 2002; Mohammad et al. 2014; Mold et al. 2019b). In general, the concentration of aluminum in fruits and vegetables depends on different factors, including the acidity of the soil, the water used for irrigation, and the plant variety. As a result of these factors, Hardisson et al. (2017) have shown that fruits and vegetables from different origins have different aluminum content. For example, it was reported that an average aluminum content of 32.8 mg/kg was found in bananas from Spain, while bananas from the USA have an aluminum content of 0.4 mg/kg (Soni et al. 2001; González-Weller et al. 2010). Besides, some plants tend to accumulate aluminum more in their roots while others accumulate aluminum more in their leaves (e.g., tea) (Hardisson et al. 2017). It was reported that carrots, spinach, cabbages, watercress, and squashes contain high level of aluminum (27.47 mg/kg), where the origin of these vegetables is Spain (González-Weller et al. 2010). Soni et al. (2001) have also reported that baked potato from the USA contains high aluminum content (26 mg/kg). Table 1 shows the mean aluminum content in some food groups. It illustrates that vegetables, fruits, roots and tubers, and seafood are all high in aluminum content (Hardisson et al. 2017).

**Table 1:** Mean aluminum content in some food groups (Hardisson et al. 2017

Food group	Mean Al content (mg/kg)
Drinks^a^	1.11
Eggs	1.52
Dairy products^b^	3.05
Fruits	6.84
Roots and tubers	9.6
Seafood	11.9
Vegetables	16.8

^a^Include soft drinks, alcoholic beverages, and some fruit juices

^b^Exclude processed cheese which has high aluminum content

The presence of aluminum in water and air is not restricted to natural processes. Human activities have major contribution to the existence of aluminum in air and water. Air emissions from the aluminum production process, coal combustion, mining, waste incineration, and motor vehicle exhaust all contribute to higher aluminum concentration in the air (Barabasz et al. 2002; Keith et al. 2008; Mirza et al. 2017; Mold et al. 2019b). Many studies have shown that particulate matter in urban areas has a substantial amount of aluminum from natural and human-related activities (Al-Thani et al. 2018a; Al-Thani et al. 2018b; Al-Thani et al. 2020; Roshan et al. 2019; Roshan et al. 2020).

In drinking water, aluminum concentration differs based on the source of water and whether aluminum is used in the process of water treatment (Mold et al. 2019b; Redgrove et al. 2019). For the process of coagulation-flocculation for water treatment, aluminum salts are mostly used as coagulants to lower the turbidity (Chuchu et al. 2013; Mohammad et al. 2014). Although some sources of food contain aluminum naturally, most of what we eat contains aluminum as food additives. Processed dairy products, mainly processed cheese, breakfast cereals, flour, cake, biscuit, baking powder, coffee, milk powder, table salts, bread, rice, and soft drinks, are all examples of food that are high in aluminum additives (Mohammad et al. 2014; Mold et al. 2019b). In fact, aluminum additives are utilized in food pickling and preserving processes (Mohammad et al. 2014). Examples of aluminum additives for different food products as well as the function of these additives are presented in Table 2 (Centre for Food Safety 2009).

**Table 2:** Examples of aluminum additives (Centre for Food Safety 2009)

Food	Aluminum additives	Function
Processed dairy products (mainly processed cheese)	Sodium aluminum phosphate-basic	An emulsifier
Pickles	Aluminum potassium sulfate	A firming agent
Baking powder (e.g., in cake, bread)	Aluminum sodium sulfate	A raising agent
Beverage	Sodium aluminosilicate	An anti-caking agent

A human can ingest a significant amount of aluminum not only because of the aluminum existence in food but also as a result of cooking with aluminum utensils, food packaging with aluminum foil, and food stored in aluminum cans (Crisponi et al. 2013). Cooking with aluminum utensils causes the leaching of aluminum from the utensils into the food as the utensil is heated. The study done by Mohammad et al. (2011) showed that the amount of aluminum leaching in different foods is considered unacceptable by WHO. The contact of food during food packaging with aluminum foil results in aluminum migration to food (Stahl et al. 2017). In fact, a recent study was done to show the difference between baking in aluminum foil cups and silicon cups (Hafez et al. 2018). The study showed that cakes baked in aluminum foil cups contain high aluminum levels, which is considered unacceptable according to WHO (Hafez et al. 2018). It is also significant to mention that cooking acidic food, such as tomatoes, in aluminum cookware is considered totally unsafe because aluminum from the cookware is released more into acidic foods (Crisponi et al. 2013). Therefore, humans are exposed to different aluminum concentrations from food depending on the food source, food type, cooking, and storage method (Crisponi et al. 2013).

In addition, it has been reported that milk formula for infants contains a noticeable amount of aluminum, especially soy-based milk formula, which was found to have a high level of aluminum (Crisponi et al. 2013). Recently, different research studies have focused on determining the concentration of aluminum in several brands of milk formula to investigate if the amount is negligible or intolerable for the health of infants.

As for the aluminum level in seafood, it is suggested that aqueous organisms can accumulate aluminum in their bodies due to water being contaminated with a high level of aluminum (Crisponi et al. 2013). In fact, Woodburn et al. (2011) have studied the accumulation of aluminum in freshwater crayfish and found that crayfish have stored and accumulated aluminum due to water being contaminated with aluminum.

Other anthropogenic sources of aluminum are toothpaste, vaccination, antiperspirants, and some drugs, including buffered aspirin and antacids (Crisponi et al. 2013). Currently, there is a great concern that human exposure to toxic aluminum from numerous sources is raising the potential for harmful health effects. Aluminum has been recently associated with neurotoxicity (Klotz et al. 2017). Hence, in the following sections, the paper discusses the impacts of aluminum on human health based on several cases reported in the literature.

## Impact of aluminum on human brain tissue

In recent years, researchers have detected elevated aluminum content in the brain tissue of patients with Alzheimer’s disease, autism, and epilepsy (Mold et al. 2019b).

On the 6^th^ of July 1988, in Camelford, UK, it was reported that 20 tons of aluminum sulfate were discharged into the water supply by mistake (Miller 2016b). The aluminum sulfate was destined to a tank at Lowermoor Water Treatment Works. However, it was accidentally placed into the drinking water supply, which serves the town of Camelford. This incident has raised the aluminum concentration in the water more than 500 times the allowable limit based on the European Union Legislation (Neuropathology and Applied Neurobiology 2017). As a result, a population of 20,000 people was exposed to a very high concentration of aluminum from their water supply (Neuropathology and Applied Neurobiology 2017; Mold et al. 2019a). In the following years, the UK government has requested documentation on the environmental and health impacts on the population as a consequence of the contaminated water with aluminum (Neuropathology and Applied Neurobiology 2017). The medical studies have verified a decrease in the intellectual function in the people exposed to a high level of aluminum in their water supply (Neuropathology and Applied Neurobiology 2017). In fact, a study done by Rondeau et al. (2009) concluded that a high risk of Alzheimer’s disease (AD) and dementia is greatly associated with exposure to  ≥ 0.1 mg/L aluminum level in drinking water.

After 6 years of the Camelford accident, a 49-year-old man, who was exposed to the contaminated water, started to suffer from memory loss. Five years later, his memory became much worse, and he experienced myoclonic jerks, dysphasia, and hallucinations, and by the age of 69, he died. After his death, his brain’s analysis showed that he suffered from complex neurodegenerative diseases, including AD, and revealed an elevated aluminum level in the occipital cortex of the brain (Neuropathology and Applied Neurobiology 2017).

According to Dzulfakar et al. (2011), out of 13 studies related to the high level of aluminum in drinking water, nine studies showed a correlation between AD and the high aluminum level. At the time of the Camelford accident, a similar case was for a 44-year-old woman who experienced similar symptoms (i.e., memory loss, dysphasia, and hallucinations) and died by the age of 59 (Neuropathology and Applied Neurobiology 2017). However, it was reported by the UK government that there was no proof relating to the 1988 Camelford accident with the late health impacts (Neuropathology and Applied Neurobiology 2017).

**Fig. 2:**
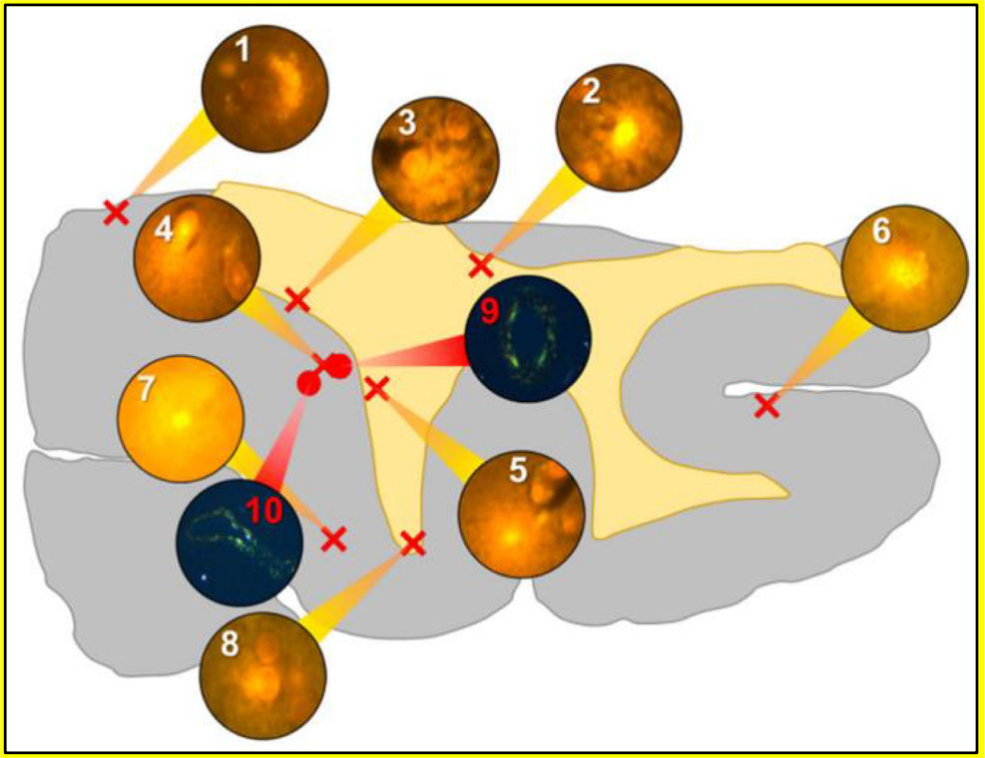
A schematic for a tissue section of the temporal lobe (Mold et al. 2019b)

Mold et al. (2019b) have recently published a paper where they revealed the third case of a 60-year-old man who was also a victim of the 1988 Camelford accident. At first, he suffered from mental problems, then from epilepsy, and then 5 years later, he died. A detailed examination of the brain revealed the presence of aluminum in several regions of the occipital lobes, temporal lobes, and hippocampal tissue. Since the hippocampus is strongly associated with epilepsy, the high level of aluminum in the hippocampus is evident that aluminum has a role in the etiology of epilepsy. In another study done by Mold et al. (2020), the quantitative examination of brain tissue for donors with autism spectrum disorder illustrated the elevated aluminum concentration, and commonly, autism and epilepsy co-occur in the same patient. The study conducted by Mold et al. 2020 does not only show clear evidence that the high content of aluminum in the brain tissue can lead to epilepsy disease, but it also demonstrates the 3^rd^ case report on the Camelford accident of water contaminated with aluminum sulfate. The fourth case reporting on the Camelford accident was for a woman who died from Cerebral amyloid angiopathy (CAA) after being exposed to the water supply contaminated with a high level of aluminum. The analysis of the brain tissue using aluminum-specific fluorescence microscopy revealed a very high aluminum level in the brain. Aluminum was abundant in the temporal lobe, parietal lobe, occipital lobe, and hippocampus. The schematic describing a tissue section of the temporal lobe in Fig. 2 identified the aluminum as orange fluorescence (in regions 1–8), whereas the apple-green birefringence (in regions 9 and 10) is evidence of CAA (Mold et al. 2019b).

Furthermore, Exley and Vickers (2014) have reported a case of a man who died from early-onset sporadic Alzheimer’s disease and was occupationally exposed to aluminum. Over 8 years, this man was inhaling the aluminum sulfate dust every day at work (Exley and Vickers 2014). He started to experience constant severe headaches and tiredness, and later on, he suffered from memory loss (Exley and Vickers 2014). The investigation of his brain tissue did not only prove the diagnosis with AD, but it also shows excessive aluminum content in the samples taken from the frontal lobe of the brain (Exley and Vickers 2014). Table 1 shows the aluminum content in 9 brain tissue samples out of 49 samples that have been studied (Exley and Vickers 2014). According to Exley and Vickers (2014), aluminum content higher than 3.50 μg/g dry weight is considered pathological. Hence, out of the nine tissue samples shown in Table 3, only samples 3 and 13 are below 3.50 μg/g dry weight.

**Table 3:** Al concentration in brain samples of AD (Exley and Thomas 2014)

Brain sample ID#	Al dry weight (μg/g)
1	7.59
3	2.06
5	4.62
13	2.26
14	4.41
19	12.97
22	4.02
23	4.81
25	12.34

In another research study, Mirza et al. (2017) have examined the brain tissue of 12 donors diagnosed with familial AD during the period 1991 to 2009. The donors included five males and seven females with an age ranging from 42 to 86 (Mirza et al. 2017). The presence of aluminum was in every sample tissue, and according to Mirza et al. (2017), “approximately 40% of tissues (57/144) had an aluminum content that was considered as pathologically-concerning (≥ 2.00 g/g dry wt.) while approximately 58% of these tissues had an aluminum content that was considered as pathologically-significant (≥ 3.00 g/g dry wt.).” The data collected from this study, along with the previous study, verify that high aluminum content in the human brain can lead to any form (sporadic or familial) of AD. In addition, from a meta-analysis done by Wang et al. (2016), it was found that chronically exposed people to aluminum from drinking water and occupation were at high risk for AD.

## Infant’s exposure to aluminum

Although aluminum is a neurotoxic substance which as discussed so far has been considered to be a major contributor to neurodegenerative diseases, infants are being exposed to this substance repeatedly through infant formula as well as vaccination. Therefore, there is an urgent need to study the long-term effects of early exposure to aluminum on infants’ developing brains. The discussion below is divided into two main parts: infant’s exposure to Al from milk formula, and infant’s exposure to Al from vaccination.

### Aluminum from milk formula

According to the WHO, it is highly recommended to exclusively breastfeed infants who are 0–6 months old (Dubascoux et al. 2015). However, only around 38% of infants (0–6 months old) in the world are exclusively breastfed (Dubascoux et al. 2015). Infant milk formulas are used as an alternative to breast milk. The main ingredient to these formulas is either cow’s milk or non-cow’s milk, mainly soya-based formula, for infants who cannot tolerate the lactose in cow’s milk. There are other ingredients in formulas to add nutritional benefits for these young children and serve as substitutes for breast milk nutrition. However, some of these formulas contain a significant amount of the toxic metal, aluminum.

**Fig. 3:**
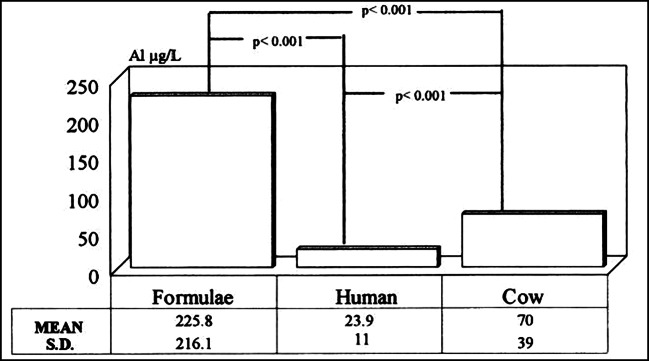
Average aluminum content in formulas, breast milk, and cow’s milk (Fernandez-Lorenzo et al. 1999)

Several studies have measured aluminum content in infant formula. Burrell and Exley (2010) have studied 15 different brands of infant formulas, including ready-made milk formulas and powdered formulas both based on cow’s milk and soya-based milk. They have found that the average ingestion of aluminum is in the range of 206 to 592 μg Al per day (depending on the type of formulas) for 6-month-old infants (Burrell and Exley 2010). The analysis method to measure the aluminum content in formulas is via the common method: transversely heated graphite furnace atomic absorption spectrometry (Redgrove et al. 2019). Milk formulas prepared from powders have higher aluminum content than ready-made milk formulas, especially soy-based milk, which has the highest ingestion of aluminum (Burrell and Exley 2010). It is widely known that soy-based formulas contain the greatest aluminum content. Chuchu et al. (2013) have found that out of 20 different milk formulas, the two soy-based formulas had the highest aluminum content. Accumulation of aluminum in soybean plants, especially for those grown in acid soils, is a major factor for the highest aluminum content in soy-based formula (Burrell and Exley 2010). Moreover, the packaging of formulas with an aluminum-based material increases the contamination of formulas. According to the study done by (Chuchu et al. 2013), they have measured the aluminum content of 30 different brands of milk formula, and all were aluminum-based packaging. Documentation of formulas with high contamination with aluminum is not a new topic, yet the values for aluminum content determined in the study done by Burrell and Exley (2010) do not significantly differ from values in older studies (Weintraub et al. 1986; Chedid et al. 1991). Burrell and Exley (2010) indicated that manufacturers are not really concerned with lowering the aluminum content in infant formula. Weintraub et al. (1986) have measured the concentration of aluminum for different brands of milk formulas from different countries (Holland, Australia, Switzerland, Canada, and the USA). They found that the concentration of aluminum in infant formulas ranged from 85 up to 5000 μg/L. Fernandez-Lorenzo et al. (1999) have determined the aluminum concentration of breast milk, infant formulas, and cow’s milk. They illustrated that the aluminum content in infant formulas (average 225.8 μg/L) was significantly higher than that in breast milk (average 23.9 μg/L), as shown in Fig. 3.

The study by Burrell and Exley was extended after 3 years to include measurements for 30 well-known brands of infant formulas in the UK. The measurements of this study are in agreement with the previous study; all 30 brands contained high values of aluminum (Chuchu et al. 2013). Hence, the authors suggested that there should be a law to obligate manufacturers of infant formula to reduce the aluminum content since after all these research results no voluntary action was taken by manufacturers (Chuchu et al. 2013).

Recently, a study was conducted to measure the aluminum content in specialized infant formulas for the first time. Pediatric clinics give specialized infant formulas to help infants who have health issues, such as poor weight gain, low birth weight (preterm and intrauterine growth restriction (IUGR) infants), and allergy (Redgrove et al. 2019). The highest aluminum content was in formulas for infants with poor weight gain (153.5–1956.3 μg/L) (Redgrove et al. 2019). The specialized formulas for preterm and IUGR had an average aluminum content in the range of 49.9–249.4 μg/L (Redgrove et al. 2019). The lowest aluminum contents were in specialized formulas for allergies and formulas with amino acid supplements (Redgrove et al. 2019). According to the authors, since some formulas had low aluminum content, this indicates that contamination with aluminum is not unavoidable (Redgrove et al. 2019). There might be ingredients that can be added to formulas to lower the aluminum content. This remarkable point by this recent study should be more investigated in the future to find out the ingredients that are successful in lowering aluminum content in milk formulas.

Although several studies have measured numerous numbers of different brands and types of infant formulas, only very few studies have elaborated on the impact of formulas contaminated with aluminum on an infant’s health (Freundlich et al. 1985).

Freundlich et al. (1985) have reported two cases of infants with renal failure and found to have high aluminum concentration accumulated in their brain even though they have not received any aluminum-containing agents (such as phosphate binder) or intravenous fluids. The first infant (weight: 3.3 kg) died at the age of 3 months and was found to have an aluminum content of 6.4 μg/g in the brain (Freundlich et al. 1985). The second infant (weight: 1.7 kg), who was a premature infant, died at the age of 1 month and was found to have an aluminum content of 47 μg/g in the brain (Freundlich et al. 1985). The first infant was treated with peritoneal dialysis, while the second infant was not treated with dialysis (Freundlich et al. 1985). Both infants were fed with the “Similac PM 60/40” milk formula (Freundlich et al. 1985). The milk formula, sterilized water (for preparation of milk), and dialysate concentrate were all analyzed (Freundlich et al. 1985). The aluminum content in  “Similac PM 60/40” milk formula was found to be 23 2 ± 60 ng/mL, whereas the sterilized water (4 ng/mL) and dialysate concentrate (3.4 ± 2.4 ng/mL) were found to have low aluminum content (Freundlich et al. 1985). Freundlich et al. (1985) concluded that the milk formula is a major contributor to the high aluminum level in infants’ brains. Hence, they recommended reducing the aluminum intake from milk formula, especially for infants with reduced renal function. According to the recent report published by the American Academy of Pediatrics (Corkins 2019), there is a great need for studies documenting the health hazards associated with the high level of aluminum present in milk formula on infants, and an urgent need for preterm infants and infants with impaired renal functions.

### Aluminum from vaccination

**Fig. 4:**
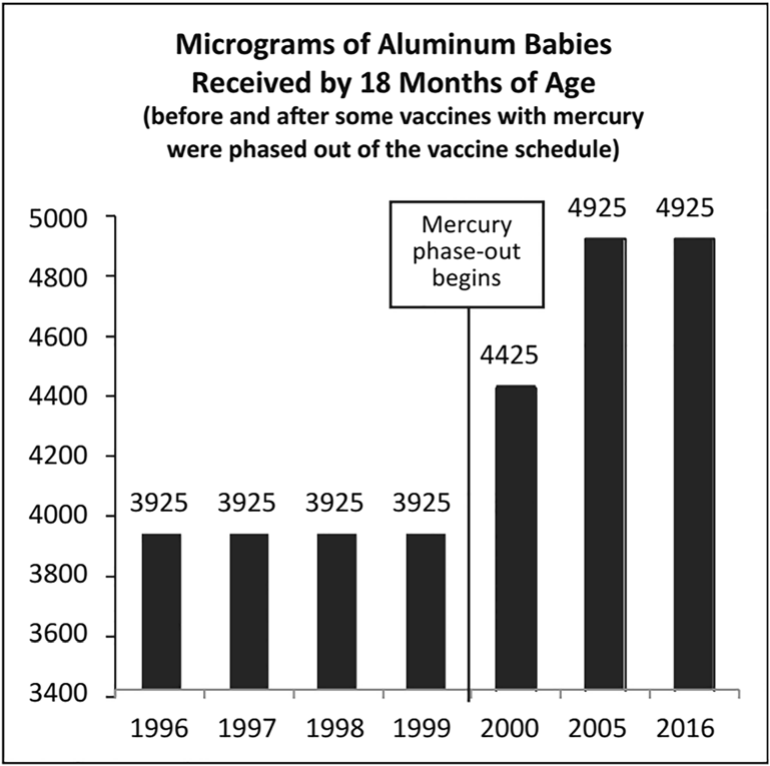
Aluminum content in vaccination (1996–2016) (Miller 2016b)

Aluminum formulations, including aluminum hydroxides and aluminum phosphate, are commonly used as adjuvants in vaccines (Baylor et al. 2002). They help enhance the efficiency of vaccination by improving the immune response to vaccine antigens (Gołoś and Lutyńska 2015). However, aluminum formulations can cause allergic responses such as redness, pain, and itchiness at the injection site, but usually, they are mild (Gołoś and Lutyńska 2015). Currently, some studies are raising the concern of vaccination containing too much of the neurotoxic substance, aluminum (Miller 2016b). McFarland et al. (2020) stated that although aluminum content in some vaccines is vital to help in functionalizing the vaccinations, the health concern associated with childhood exposure to injected aluminum is of great importance. In 2000, mercury was phased out from vaccines for children’s immunization schedule (Miller 2016b). Before 2000, infants were receiving 3925 μg of aluminum in their first 18 months. By the year 2005, infants started to receive 4925 *μg* of aluminum (i.e., 25% increase) in their first 18 months, as shown in Fig. 4. The reason behind this increase is due to the addition of six doses of aluminum-containing vaccines. Four doses were added in the year 2000 for pneumococcal, and another two doses were added in the year 2005 for hepatitis A (Miller 2016b).

In addition, in 2011, the Center for Disease Control and Prevention (CDC) recommended pregnant women to take vaccination with aluminum content (Miller 2016b). Aluminum accumulates in the fetal tissue since it passes through the placenta (Miller 2016b). This means that infants are receiving a considerable amount of aluminum even before being born (Miller 2016b). The aluminum contents in vaccines received from birth to 18 months are illustrated in Table 4 (Miller 2016b).

**Table 4:** Aluminum content in vaccination (birth-18 months) (Miller 2016b)

Vaccine*	Aluminum content	Vaccine schedule
Hep B	250 μg × 3 doses	Birth, 2, 6 months
DTaP	625 μg × 4 doses	2, 4, 6, 15 months
PCV	125 μg × 4 doses	2, 4, 6, 12 months
Hib	225 μg × 3 doses	2, 4, 12 months
Hep A	250 μg × 2 doses	12, 18 months

*Hep B hepatitis B, *DTaP* diphtheria, tetanus, and pertussis, *PCV* pneumococcal, *Hip* hemophilus influenzae type b, *Hep A* hepatitis A

The injection with aluminum adjuvants repeatedly during the critical period where the child’s brain is developing is of significant concern. Tomljenovic and Shaw (2012) argued that children are at high risk of Al adjuvants’ adverse reactions. According to the authors, a neonate has an immature renal system that lowers the ability to effectively eliminate the neurotoxic Al from the body (Tomljenovic and Shaw 2012). They stated that there is an urgent need for evaluating the potential health impact of vaccines (Tomljenovic and Shaw 2012). The US Food and Drug Administration (FDA) has established in 2004 the Al limit from injection sources for premature infants to be < 4–5 μg/kg bw/day and confirmed that above this level, there is a potential for bone and central nervous system (CNS) toxicity (Food and Drug Administration (FDA) Department of Health and Human Services 2004). However, infants are receiving 14.7–18.4 times more than this FDA limit (4–5) from only one dose of Hep B vaccine, as illustrated in Table 4 (73.5 μg Al/kg bw/day for a newborn with 3.4 kg bw) (Tomljenovic and Shaw 2012). Tomljenovic and Shaw 2011) declared that “vaccine-derived aluminum has a much greater potential to induce neurological damage than that obtained through diet, even in those with effective renal function.” Similarly, Dórea (2020) stated that “100% of metabolic active Al” is left from typhoid conjugate vaccine shots in the 1^st^ 6 months. Hence, there is a great risk for long-term harmful effects on the neurological system from using aluminum adjuvants in vaccination (Tomljenovic and Shaw 2011).

In 1997, Bishop et al. (1997) had studied and compared the effect of standard intravenous-feeding solutions (with aluminum content 25 μg/deciliter) and aluminum-depleted intravenous-feeding solutions (with aluminum content 2.2 μg/deciliter) on preterm infants. The study revealed that the Bayley Mental Development Index was lower for preterm infants who received standard intravenous-feeding solutions (Bishop et al. 1997). However, Bishop et al. (1997) stated that “the effect of aluminum exposure was dose-related” since infants who received standard intravenous-feeding solutions for more than 10 days are the ones who showed a major decline in neurological development by the age of 18 months.

More recently, Fanni et al. (2014) stated that children, infants, and especially premature infants are at great risk of aluminum toxicity since they are still undergoing growth and development stages. In fact, there are three infants’ groups at the greatest risk of aluminum toxicity: premature infants who need large doses of phosphate and calcium for bone mineralization, infants receiving parenteral nutrition for a long period, and infants with reduced kidney function (Fanni et al. 2014). Fanni et al. (2014) have reviewed the possible health consequence of aluminum exposure on fetuses and infants. During pregnancy, many pregnant women take anti-reflux medicines (e.g., antacids) containing aluminum (Fanni et al. 2014). According to the authors, experiments conducted on pregnant rats revealed that “oral exposure during pregnancy can produce significant changes in the tissue distribution of multiple essential trace elements, with possible consequences on fetal metabolism” (Fanni et al. 2014). It was suggested that anti-reflux medicines should be consumed with precautions and limitations to avoid aluminum’s fetal toxicity (Fanni et al. 2014).

As for infants receiving parenteral nutrition, they are at a very high risk of aluminum toxicity since all of the ingredients for parenteral nutrition contain a high level of aluminum (Corkins 2019). Although the renal system takes care of clearing aluminum from the body, a high level of aluminum for a long duration is a great health concern. In addition, infants with impaired renal function, especially preterm infants, cannot excrete this large excess amount of aluminum, making them more vulnerable to aluminum toxicity (Fanni et al. 2014). According to Corkins (2019), if aluminum accumulates to a high level in the bone, there is a great risk of osteomalacia development. In fact, the study by Bishop et al. (1997) was later developed by Fewtrell et al. (2009) via following up on the same infants at the age of 13–15 years old to investigate the effect of parenteral nutrition on the bone. It was shown that adolescence who have earlier received the standard (higher aluminum level) parenteral nutrition are the ones who have lower bone mineral content (BMC) of the lumbar spine and hip (Fewtrell et al. 2009). This means that they have the potential of developing osteomalacia and fracture risk (Fewtrell et al. 2009). Therefore, it was concluded that there is a long-term effect on bone associated with receiving aluminum from standard parenteral nutrition solutions.

## Dermal exposure to aluminum: antiperspirants and cosmetics

Antiperspirants contain aluminum since they can block sweat secretion by plugging the gland (Flarend et al. 2001). Aluminum is not only present in antiperspirants; in fact, it is one of the components in many cosmetics products such as makeup, creams, and mud from the Dead Sea, as illustrated in Table 5 (Borowska and Brzóska 2015).

**Table 5:** Aluminum concentration in some cosmetics (Borowska and Brzóska 2015)

Products	Al content (mg/kg)
Eye shadow	20,000–50,000
Mascara	117–20,000
Lipsticks	14.2–27,032
Lip glosses	0.415–10,536
Foundations, compact powders	33.26–18,661.5
Creams	15.31–62.17
Muds from the Dead Sea	4500–7900
Hand creams	5400–8500
Facial mask	170–650
Henna	142.1
Kohl	56.75–1009.3

Therefore, the human body is dermally exposed to Al by applying antiperspirants and cosmetic products (de Ligt et al. 2018). Specifically, aluminum chlorohydrate is an important ingredient present in many antiperspirant brands to treat hyperhidrosis (de Ligt et al. 2018). The frequent use of these items means that Al is accumulated in the body (Borowska and Brzoska 2015). As mentioned previously, Al is a toxic metal which has been linked to AD and other neurological disorder, and in the long-term contributes to bone toxicity. Hence, measuring the concentration of Al in antiperspirants and cosmetics and how much absorbed by the skin is of great necessity to ensure safe dermal use of these products. Unfortunately, there are only very few studies concerned with dermal exposure to Al (Corkins 2019; de Ligt et al. 2018; Freundlich et al. 1985; Weintraub et al. 1986).

Guillard et al. (2004) have reported a case for a 43-year-old woman who experienced pain in her bones and excessive fatigue as a result of overexposure to Al from antiperspirant. The woman applied around 1 g of antiperspirant cream (contains 20% of aluminum chlorohydrate) on each of her underarms on a daily basis for 4 years. It took 3 months for Al in urine to reach the reference level and 8 months in plasma. According to the patient, the pain in the bone stopped after 8 months of stopping the use of antiperspirants (Guillard et al. 2004).

It is vital to mention that the patient who had normal kidney function, was not occupationally exposed to Al, and did not take any antacids that contain Al. The concentration of aluminum in urine was 46.1 μg/24 h whereas the normal concentration is 29.7 μg/24 h (Guillard et al. 2004). As a result, Guillard et al. (2004) suggested that the main reason for this overexposure is the continuous use of aluminum-containing-antiperspirant for a long period.

## Conclusion

Aluminum (Al) is the most abundant metal on earth, and it exists in nature with other elements as different compounds. There are both natural and anthropogenic sources for Al. Natural sources include rocks, soil, air, water, acid rains, and some plants (e.g., tea). The presence of Al in air and water is not only natural but is also due to human activities, such as air pollution and the water treatment process, which includes the use of Al as coagulants. In addition, Al exists as a food additive in many kinds of food and found in packaging, storing, and utensils.

Since humans are exposed to Al from numerous sources, especially anthropogenic sources, on a daily basis, there is a great concern that this exposure is harmful to human health. First, Al has been associated with neurotoxicity. Recently, elevated Al level has been detected in the brain tissue for patients with AD and other neurodegenerative diseases. Second, studies have shown that milk formulas contain an intolerable amount of Al; hence, infants are being overexposed to this toxic metal. Although some of these studies are old, recent studies measuring Al content indicate that no action was taken to lower the Al level. Third, recent studies are raising the concern of infants’ vaccination containing too much Al.

Moreover, intravenous-feeding solutions compose of high Al concentration. Infants, especially premature ones and infants with renal failure, are at higher risk for accumulation of Al in the brain and bones which is a potential for CNS and bone toxicity in the long term. Some people may argue that aluminum adjuvants have shown to be effective, and the concern about their toxicity has not been proven. However, infants are at vital stages of developing and growing; hence, higher precautions should be taken by assessing the potential health risk associated with vaccines as well as intravenous-feeding solutions. Finally, humans are dermally exposed to Al through the frequent use of antiperspirants and some cosmetics for a long period. However, there are not enough studies proving the long-term health effect of antiperspirants.

## Recommendations

Based on the previous studies and this review paper, we highly recommend the following:

1. The cases reported in the literature have linked the high exposure of aluminum to AD and demonstrated the existence of high aluminum content in the brain tissues. However, since these cases are not representative of the total population, it is recommended that more research studies are needed to confirm the direct correlation between high-concentration/long-term exposure to Al and neurodegenerative diseases, mostly AD.
2. To the best of our knowledge, the exact mechanism(s) of how the aluminum content in brain tissue results in the development of neurodegenerative diseases have not been fully understood/known. More studies should concentrate on understanding the exact mechanism and report the scientific details on how the existence of high aluminum content in the brain causes the development of neurodegenerative diseases.
3. Although several studies have measured and illustrated that many types/brands of infants’ milk formulas contain a significant amount of Al, studies are required to substantiate the long-term health impact associated with feeding infants these milk formulas.
4. More studies are needed to evaluate aluminum adjuvants’ potential health impact on infants and especially premature ones.
5. Studies should be done to measure the Al content in different antiperspirants, determine how much absorbed by the skin and normally excreted by the body, and investigate the long-term health effect associated with the usage of antiperspirants.

## Data Availability

Not applicable.

## References

[CR1] Al-Thani H, Koç M, Fountoukis C, Isaifan RJ (2020). Evaluation of particulate matter emissions from non-passenger diesel vehicles in Qatar. J Air Waste Manag Assoc.

[CR2] Al-Thani H, Koç M, Isaifan RJ (2018). A review on the direct effect of particulate atmospheric pollution on materials and its mitigation for sustainable cities and societies. Environ Sci Pollut Res.

[CR3] Al-Thani H, Koç M, Isaifan RJ (2018). Investigations on deposited dust fallout in urban Doha: characterization, source apportionment and mitigation. Environ Ecol Res.

[CR4] Barabasz W, Albinska D, Jaskowska M, Lipiec J (2002). Ecotoxicology of Aluminium. Pol J Environ Stud.

[CR5] Baylor NW, Egan W, Richman P (2002). Aluminum salts in vaccines — US perspective. Vaccine.

[CR6] Bishop NJ, Ruth M, Chir B, Day P, Lucas A (1997). Aluminum neurotoxicity in preterm infants receiving intravenous-feeding solutions. N Engl J Med.

[CR7] Borowska S, Brzóska MM (2015). Metals in cosmetics : implications for human health. J Appl Toxicol.

[CR8] Burrell S-a M, Exley C (2010) There is (still) too much Aluminium in infant formulas. BMC Pediatr 10:2–5 10.1186/1471-2431-10-63 PMC2939626 20807425

[CR9] Centre for Food Safety (2009) Aluminium in food. http://scholar.google.com/scholar?hl=en&btnG=Search&q=intitle:Aluminium+in+food#0

[CR10] Chedid F, Fudge A, TeubnerR J, James SL, Simmer K (1991). Aluminium absorption in infancy. J Paediatr Child Health.

[CR11] Chuchu N, Patel B, Sebastian B, Exley C (2013). The Aluminium content of infant formulas remains too high. BMC Pediatr.

[CR12] Corkins MR (2019) Aluminum effects in infants and children. Am Acad Pediatr 144. 10.1542/peds.2019-3148 10.1542/peds.2019-3148 31767714

[CR13] Crisponi G, Fanni D, Gerosa C, Nemolato S, Nurchi VM, Crespo-Alonso M, Lachowicz JI, Faa G (2013). The meaning of Aluminium exposure on human health and Aluminium-related diseases. Biomol Concepts.

[CR14] Dórea JG (2020). Neurotoxic effects of combined exposures to aluminum and mercury in early life ( infancy). Environ Res.

[CR15] Dubascoux S, Nicolas M, Rime CF, Payot JR, Poitevin E (2015). Simultaneous determination of 10 Ultratrace elements in infant formula, adult nutritionals, and Milk products by ICP/MS after pressure digestion: single-laboratory validation. J AOAC Int.

[CR16] Dzulfakar MA, Shaharuddin MS, Muhaimin AA, Syazwan AI (2011). Risk assessment of aluminum in drinking water between two residential areas. Water.

[CR17] Exley C, Vickers T (2014) Elevated brain Aluminium and early onset Alzheimer's disease in an individual occupationally exposed to Aluminium : a case report. J Med Case Rep 8(1):1–3. 10.1186/1752-1947-8-41 10.1186/1752-1947-8-41 PMC3923550 24513181

[CR18] Fanni D, Ambu R, Gerosa C, Nemolato S, Iacovidou N, Van Eyken P, Fanos V, Zaffanello M, Faa G (2014). Aluminum exposure and toxicity in neonates: a practical guide to halt aluminum overload in the prenatal and perinatal periods. World J Pediatr.

[CR19] Fernandez-Lorenzo JR, Cocho JA, Rey-Goldar ML, Couce M, Fraga JM (1999). Aluminum contents of human milk, cow’s milk, and infant formulas. J Pediatr Gastroenterol Nurtr.

[CR20] Fewtrell MS, Bishop NJ, Edmonds CJ, Isaacs EB, Lucas A (2009). Aluminium exposure from intravenous feeding solutions and later bone health: 15 year follow-up of a randomised trial in preterm infants. Pediatrics.

[CR21] Flarend R, Bin T, Elmore D, Hem SL (2001). A preliminary study of the dermal absorption of Aluminium from antiperspirants using Aluminium-26. Food Chem Toxicol.

[CR22] Food and Drug Administration (FDA) Department of Health and Human Services (2004) Aluminum in large and small volume parenterals used in total parenteral nutrition. https://www.govinfo.gov/content/pkg/CFR-2005-title21-vol4/pdf/CFR-2005-title21-vol4-sec201-323.pdf

[CR23] Freundlich M, Zilleruelo G, Abitbol C, Strauss J (1985) Infant formula as a cause of Aluminium toxicity in neonatal Uraemia. Lancet:527–529 10.1016/s0140-6736(85)90463-5 2863545

[CR24] Gołoś A, Lutyńska A (2015). Aluminium-Adjuvanted vaccines-a review of the current state of knowledge. Przegl Epidemiol.

[CR25] González-Weller D, Gutiérrez ÁJ, Rubio C, Revert C, Hardisson A (2010). Dietary intake of aluminum in a Spanish population (Canary Islands). J Agric Food Chem.

[CR26] Guillard O, Fauconneau B, Olichon D, Dedieu G, Deloncle R (2004). Hyperaluminemia in a woman using an aluminum-containing antiperspirant for 4 years. Am J Med.

[CR27] Hafez HHA, Abd El-Salam HM, Huseen GH (2018). Studies on the effect of aluminum, aluminum foil and silicon baked cups on aluminum and silicon migration in cakes. Egypt J Agric Res.

[CR28] Hardisson A, Revert C, González-Weller D, Gutiérrez Á, Paz S, Rubio C (2017). Aluminium exposure through the diet. Food Sci Nutr.

[CR29] Igweze ZN, Ekhator OC, Nwaogazie I, Orisakwe OE (2020). Public health and Paediatric risk assessment of Aluminium, arsenic and mercury in infant formulas marketed in Nigeria. Sultan Qaboos Univ Med J.

[CR30] Keith S, Jones D, Rosemond Z, Ingerman L, Chappell L (2008) Potential for human exposure. Toxicological Profile for Aluminum:175–227

[CR31] Klotz K, Weistenhöfer W, Neff F, Hartwig A, Van Thriel C, Drexler H (2017). The health effects of aluminum exposure. Dtsch Arztebl Int.

[CR32] de Ligt R, van Duijn E, Grossouw D, Bosgra S, Burggraaf J, Windhorst A, Peeters PAM, van der Luijt GA, Alexander-White C, Vaes WHJ (2018). Assessment of dermal absorption of aluminum from a representative antiperspirant formulation using a Al microtracer approach. Clin Transl Sci.

[CR33] McFarland G, La Joie E, Thomas P, Lyons-Weiler J (2020). Acute exposure and chronic retention of aluminum in three vaccine schedules and effects of genetic and environmental variation. J Trace Elem Med Biol.

[CR34] Miller NZ (2016). Aluminum. Miller’s review of critical vaccine studies: 400 important scientific papers summarized for parents and researchers.

[CR35] Miller NZ (2016). Aluminum in childhood vaccines is unsafe. J Am Physicians Surg.

[CR36] Mirza A, King A, Troakes C, Exley C (2017) Aluminium in brain tissue in familial Alzheimer' disease. J Trace Elem Med Biol 40:30–36. 10.1016/j.jtemb.2016.12.001 10.1016/j.jtemb.2016.12.001 28159219

[CR37] Mohammad FS, Al Zubaidy EAH, Bassioni G (2011). Effect of aluminum leaching process of cooking wares on food. Int J Electrochem Sci.

[CR38] Mohammad FS, Al Zubaidy IAH, Bassioni G (2014). A comparison of aluminum leaching processes in tap and drinking water. Int J Electrochem Sci.

[CR39] Mold M, Cottle J, Exley C (2019). Aluminium in brain tissue in epilepsy : a case report from Camelford. Int J Envriron Res Public Health.

[CR40] Mold M, Cottle J, King A, Exley C (2019). Intracellular Aluminium in inflammatory and glial cells in cerebral amyloid Angiopathy: a case report. Int J Environ Res Public Health.

[CR41] Mold M, Linhart C, Gómez-Ramírez J, Villegas-Lanau A, Exley C (2020). Aluminum and amyloid-β in familial Alzheimer’s disease. J Alzheimers Dis.

[CR42] Neuropathology and Applied Neurobiology (2017) Unusual neuropathological features and increased brain aluminium in a resident of Camelford, UK. Scientific Correspondence:1–5. 10.1111/nan.12417 10.1111/nan.12417 28603852

[CR43] Redgrove J, Rodriguez I, Mahadevan-bava S, Exley C (2019). Prescription infant formulas are contaminated with Aluminium. Int J Envriron Res Public Health.

[CR44] Rondeau V, Jacqmin-Gadda H, Commenges D, Helmer C, Dartigues JF (2009). Aluminum and silica in drinking water and the risk of Alzheimer’s disease or cognitive decline: findings from 15-year follow-up of the PAQUID cohort. Am J Epidemiol.

[CR45] Roshan DR, Koc M, Abdallah A, Martin-Pomares L, Isaifan R, Fountoukis C (2020). UV index forecasting under the influence of desert dust: evaluation against surface and satellite-retrieved data. Atmosphere.

[CR46] Roshan DR, Koc M, Isaifan R, Shahid MZ, Fountoukis C (2019). Aerosol optical thickness over large urban environments of the Arabian peninsula-speciation, variability, and distributions. Atmosphere.

[CR47] Soni MG, White SM, Gary Flamm W, Burdock GA (2001). Safety evaluation of dietary aluminum. Regul Toxicol Pharmacol.

[CR48] Stahl T, Falk S, Rohrbeck A, Georgii S, Herzog C, Wiegand A, Hotz S, Boschek B, Zorn H, Brunn H (2017) Migration of aluminum from food contact materials to food—a health risk for consumers? Part I of III: exposure to aluminum, release of aluminum, tolerable weekly intake (TWI), toxicological effects of aluminum, study design, and methods. Environ Sci Eur 29. 10.1186/s12302-017-0116-y 10.1186/s12302-017-0116-y PMC5388732 28458989

[CR49] Tomljenovic L, Shaw CA (2011). Aluminum vaccine adjuvants: are they safe?. Curr Med Chem.

[CR50] Tomljenovic L, Shaw CA (2012). Mechanisms of aluminum adjuvant toxicity and autoimmunity in pediatric populations. Lupus.

[CR51] Wang Z, Wei X, Yang J, Suoa J, Chen J, Liu X, Zhao X (2016). Chronic exposure to aluminum and risk of Alzheimer’s disease : a meta-analysis. Neurosci Lett.

[CR52] Weintraub R, Hams G, Meerkin M, Rosenberg AR (1986). High Aluminium content of infant milk formulas. Arch Dis Child.

[CR53] Woodburn K, Walton R, Mccrohan C, White K (2011). Accumulation and toxicity of Aluminium-contaminated food in the freshwater crayfish, Pacifastacus Leniusculus. Aquat Toxicol.

[CR54] World Health Organization (2003) Aluminium in drinking-water. 2

[CR55] Zhou J, Zhu L, Zhou T, Xin Z, Wu L, Luo Y, Christie P, Christie P (2020). Aluminum toxicity decreases the Phytoextraction capability by cadmium/zinc Hyperaccumulator sedum Plumbizincicola in acid soils. Sci Total Environ.

